# A Dynamic Attitude Measurement System Based on LINS

**DOI:** 10.3390/s140916082

**Published:** 2014-08-29

**Authors:** Hanzhou Li, Quan Pan, Xiaoxu Wang, Juanni Zhang, Jiang Li, Xiangjun Jiang

**Affiliations:** 1 School of Automation, Northwestern Polytechnical University, Xi'an 710072, China; E-Mails: Quanpan@nwpu.edu.cn (Q.P.); woyaofly@163.com (X.W.); 2 The Sixteenth Research Institute of CASC, Xi'an 710100, China; E-Mails: zhangjuanni221@163.com (J.Z.); jxj97011311@163.com (X.J.); 3 Northwest Institute of Mechanical & Electrical Engineering, Xian Yang 712099, China; E-Mail:lixk@sina.com

**Keywords:** laser inertial navigation system, attitude reference, dynamic attitude measurement, coning error, time synchronization, phase compensation

## Abstract

A dynamic attitude measurement system (DAMS) is developed based on a laser inertial navigation system (LINS). Three factors of the dynamic attitude measurement error using LINS are analyzed: dynamic error, time synchronization and phase lag. An optimal coning errors compensation algorithm is used to reduce coning errors, and two-axis wobbling verification experiments are presented in the paper. The tests indicate that the attitude accuracy is improved 2-fold by the algorithm. In order to decrease coning errors further, the attitude updating frequency is improved from 200 Hz to 2000 Hz. At the same time, a novel finite impulse response (FIR) filter with three notches is designed to filter the dither frequency of the ring laser gyro (RLG). The comparison tests suggest that the new filter is five times more effective than the old one. The paper indicates that phase-frequency characteristics of FIR filter and first-order holder of navigation computer constitute the main sources of phase lag in LINS. A formula to calculate the LINS attitude phase lag is introduced in the paper. The expressions of dynamic attitude errors induced by phase lag are derived. The paper proposes a novel synchronization mechanism that is able to simultaneously solve the problems of dynamic test synchronization and phase compensation. A single-axis turntable and a laser interferometer are applied to verify the synchronization mechanism. The experiments results show that the theoretically calculated values of phase lag and attitude error induced by phase lag can both match perfectly with testing data. The block diagram of DAMS and physical photos are presented in the paper. The final experiments demonstrate that the real-time attitude measurement accuracy of DAMS can reach up to 20″ (1σ) and the synchronization error is less than 0.2 ms on the condition of three axes wobbling for 10 min.

## 1. Introduction

An inertial navigation system (INS) has unparalleled advantages in measuring the motion parameters of vehicles and can output the moving vehicle's attitude, velocity, and displacement in a real-time and autonomous way. Therefore, it is widely applied in military weapon systems such as spacecraft, missiles, airplanes, ships, advanced ground vehicles, and so on. At present, the focus of INS development is aimed at producing low cost, high precision and miniature size units [1–4]. Besides its applications in inertial navigation and guidance, as an attitude sensor, INS can also provide attitude data of vehicles for other equipment. For example, the attitude reference for synthetic aperture radar (SAR) or lidar can be offered by a position orientation system (POS), the attitude data for the servo control system of antiaircraft guns can be measured by INS, the accuracy of servo turntables or other attitude measurement systems can be verified by higher precision INS, and so on. Among all such applications, INS is unexceptionally required to output high precision attitude data of the base at each sampling time. Therefore, attitude measurement is considered as a major application of INS.

An attitude heading reference system (AHRS) and an attitude determination system (ADS) can perform real-time measurements of a vehicle's attitude. Both of them are widely used in all kinds of vehicles under water, on the ground and in the air [5–8]. AHRS and ADS that are made up of different types of gyros differ enormously in accuracy. For example, in [5], an ADS contains a MicroElectro Mechanical System (MEMS) gyro and magnetic compass, by means of which the accuracy of static horizontal attitude reaches 0.1°, while the dynamic accuracy drops to 2°. Sun *et al.* [6] employs a fiber optic gyro (FOG) to form ADS and takes Phins as reference for a ship test. As a result, the accuracy of horizontal attitude is improved to 0.05° and that of heading to 0.1°. Same famous companies have developed popular high precision AHRS products based on RLG. The heading and level attitude accuracy of Honeywell AH-2100 Super AHRS are 1° and 0.15°, respectively [9]. Another high precision AHRS, the Sigma 40, was developed by the Sagem Company of France; its heading and horizontal attitude accuracy are 3′ and 1′, respectively [10]. It can be found that the accuracy of all products mentioned above just reaches several minutes of arc at most, and the dynamic accuracy is lower than the static accuracy. It is necessary to develop a kind of higher dynamic attitude measurement which is needed in some fields such as high precision surveying and high attitude reference.

Three problems need to be solved when an INS is applied to measuring high precision and real-time attitude in dynamic maneuver environments. Firstly, due to intrinsic attributes of inertial elements and characteristics of navigation algorithm, vehicle motion will stimulate extra navigation errors called dynamic errors [11,12], whose magnitudes are relevant to the categories of inertial instruments, configurations of INS, navigation algorithm and the characteristics of vehicle motion. Reduction or compensation of dynamic errors is a hot research topic in the inertial navigation field [13–15]. Secondly, because the measured vehicle is in motion, the correspondence relationship between measurement data and vehicle motion can be indicated only when the data from INS have a time stamp or some kind of time synchronization mechanism. This is analogous to time synchronization adopted in integrated Global Position System (GPS)/INS navigation. In a loose couple model, both GPS and INS can output latitude, longitude and velocities of the vehicle, but because of the differences between these two systems, for example, the sampling moment and the sampling frequency, fusion of the data from the two systems must depend on a specific synchronization mechanism to determine the fusion moment [16]. Thirdly, as a motion sensing sensor, INS has its transfer function in frequency domain, and thus inevitably leads to a phase lag in the output data because of mechanical structure, gyro, data acquisition and procession issues. The system phase lag in the frequency domain can produce a time delay in the time domain, therefore, it will cost a lot of dynamic attitude measurement accuracy of the INS. In other words, the usual INS is not suitable for high precision dynamic attitude measurement if time synchronization and phase compensation are not considered. For the above mentioned time synchronization realized by GPS/INS integrated navigation, only the errors of synchronization between attitude updating clock of INS and pulses per second (PPS) are taken into account, and the dynamic errors induced by phase lag are not concerned. In [17], the issue of time-space consistency among INS, differential GPS (DGPS) and lidar is put forward and when an onboard POS is employed to provide attitude information for lidar, the article clearly states that the installation position of sensors, time synchronization errors and time delay would result in measurement errors, yet quantitative analysis of errors induced by system delay or solutions is not mentioned. In [18], it is found that there exists a phase difference between POS data and those from lidar when the data from an onboard lidar and POS are fused, and the article comes up with a way of eliminating this type of delay by changing universal time coordinated (UTC) time stamp of attitude information from the POS. However, as a matter of fact, this is a rough solution, because generally POS outputs data at a frequency of 100 ∼ 200 Hz and a time stamp resolution ratio is 5∼10 ms. If the airplane moves with a velocity of 200 m/s and at an altitude of 600 m, one missed frame will cause the ground positioning error to be 1 ∼ 2 m, which is unacceptable in high precision surveying and mapping applications [17].

This paper comes up with a pioneering solution by developing a high precision real-time dynamic attitude measurement system (DAMS) based on a LINS. A novel FIR filter is designed in the paper, which has three notches to simultaneously aim to the dither frequencies of three RLGs. A theorem to describe the maximum attitude measurement error induced by phase lag in dynamic attitude measurement and its proof are presented for the first time. A special synchronization mechanism is put forward to solve the problems of synchronization and phase compensation together by taking advantage of the complex programmable logic device (CPLD) in navigational computer. A synchronization mechanism verification method is raised to demonstrate the correctness of theoretical expression by means of single-axis turntable and laser interferometer. At last, the DAMS performance test results are shown that the dynamic attitude measurement accuracy is up to 20″ (1σ) under three axes wobbling test for 10 min.

## 2. Coning Errors Compensation

The strap-down inertial navigation system (SINS) isolates angular motion of the base by a mathematics platform. Constrained by the inertial element error itself, sampling rates and bandwidth of SINS, the mathematical platform of SINS is unable to eliminate completely the error induced by dynamic maneuver environments, which therefore brings about coning errors in attitude updating calculations and further causes attitude errors to increase linearly with time. As a result, coning errors are regarded as an error source severely affecting the dynamic accuracy of SINS. As one of the most rigorous test methods for checking dynamic error of SINS [19], the multiple-axis wobbling test (to excite INS sinusoidal movement using a turntable or wobbler is called a wobbling test) has become the research focus [20,21]. In this paper, the coning errors are decreased by optimizing the parameters from the perspective of attitude updating algorithm and attitude updating frequency. Wobbling tests to show the improvements achieved with the optimal methods are presented.

### 2.1. Coning Errors Compensation Algorithm by Using One Previous Sample

Optimal multi-sample algorithms [19] are widely adopted in SINS attitude updating. To further improve compensation accuracy, it is put forward that the previous *P* samples can be used to reduce the coning errors in current attitude updating period. A new *N* samples equivalent rotating vector $\Delta \vec{\hat{\Phi }}$ can be obtained as follows [21]:

(1) $$\Delta \vec{\hat{\Phi }}=\Delta \vec{\theta }+\sum_{i=1}^{N-1}K_{i}\left(\Delta {\vec{\theta }}_{i}\times \Delta {\vec{\theta }}_{N}\right)+\sum_{j=1-p}^{0}K_{j}\left(\Delta {\vec{\theta }}_{j}\times \Delta {\vec{\theta }}_{N}\right)$$

where, *K_i_* and *K_j_* are undetermined coefficients, $\Delta \vec{\theta_{\iota }}$ is *i^th^* gyro samples in the current attitude updating period, $\Delta \vec{\theta_{N}}$ denotes *N^th^* gyro samples in the current attitude updating period, $\vec{\theta }=\sum_{i=1}^{N}\Delta \vec{\theta_{\iota }}$ represents the incremental angle accumulated by gyros over the current attitude updating interval. The angular rate ω⃗ is supposed as a constant in a sampling period, and then we have:

(2) $$\begin{array}{ll} \Delta {\vec{\theta }}_{j} & \begin{array}{cc} =\int_{t+{\left(j-1\right)}^{*}h/N}^{t+j^{*}h/N}\vec{\omega }\left(\tau \right)d\tau & \text{j}=1-P,2-P,\ldots ,-1,0 \end{array}\\ & =\left[\begin{array}{c} -\frac{2}{N}\left(\omega h\right)\sin^{2}\left(\frac{\phi }{2}\right)\\ -2\sin\left(\phi \right)\sin\left(\frac{\omega h}{2N}\right)\sin\left[\omega \left(t+\frac{2j-1}{2N}h\right)\right]\\ 2\sin\left(\phi \right)\sin\left(\frac{\omega h}{2N}\right)\cos\left[\omega \left(t+\frac{2j-1}{2N}h\right)\right] \end{array}\right] \end{array}$$

From the coning error definition, we obtain:

(3) $$\begin{array}{ll} \phi_{ɛ} & =\Delta \phi_{1}-\Delta {\hat{\phi }}_{1}\\ & =-\frac{\phi^{2}}{2}\sin\left(N\lambda \right)+\frac{\phi^{2}}{2}N\lambda -\frac{\phi^{2}}{2}[4K_{1-P}\left(1-\cos\lambda \right)\sin\left(\left(N-1+P\right)\lambda \right)+\\ & 4K_{2-P}\left(1-\cos\lambda \right)\sin\left(\left(N-2+P\right)\lambda \right)+\cdots +4K_{N-1}\left(1-\cos\lambda \right)\sin\lambda ]\\ & =\frac{\phi^{2}}{2}[-\sin\left(N\lambda \right)+N\lambda -K_{1-P}\left(4\sin\left(\left(N-1+P\right)\lambda \right)-2\sin\left(\left(N+P\right)\lambda \right)-2\sin\left(\left(N-2+P\right)\lambda \right)\right)-\\ & K_{2-P}\left(4\sin\left(\left(N-2+P\right)\lambda \right)-2\sin\left(\left(N-1+P\right)\lambda \right)-2\sin\left(\left(N-3+P\right)\lambda \right)\right)-\ldots -\\ & K_{N-1}\left(4\sin\left(\lambda \right)-2\sin\left(2\lambda \right)\right)] \end{array}$$

Applying a Taylor series expansion to the right of Equation (3), we have:

(4) $$\phi_{ɛ}=\frac{\phi^{2}}{2}\left[\begin{array}{cccc} -\frac{{\left(N\lambda \right)}^{3}}{3!} & \frac{{\left(N\lambda \right)}^{5}}{5!} & -\frac{{\left(N\lambda \right)}^{7}}{7!} & \cdots \end{array}\right]\left(F\cdot \left[\begin{array}{c} K_{1-P}\\ K_{2-P}\\ \vdots \\ K_{0}\\ K_{1}\\ K_{2}\\ \vdots \\ K_{N-1} \end{array}\right]-\left[\begin{array}{c} 1\\ 1\\ \vdots \\ 1\\ 1\\ 1\\ \vdots \\ 1 \end{array}\right]\right)$$

where:

$$\begin{array}{l} \text{F}=\left[\begin{array}{cccccccc} A_{1,1-P} & A_{1,2-P} & \cdots & A_{1,0} & A_{1,1} & A_{1,2} & \cdots & A_{1,N-1}\\ A_{2,1-P} & A_{2,2-P} & \cdots & A_{2,0} & A_{2,1} & A_{2,2} & \cdots & A_{2,N-1}\\ \vdots & \vdots & \cdots & \vdots & \vdots & \vdots & \cdots & \vdots \end{array}\right]\\ A_{i,j}=\frac{-4{\left(N-j\right)}^{\left(2i+1\right)}+2{\left(N-j+1\right)}^{\left(2i+1\right)}+2{\left(N-j-1\right)}^{\left(2i+1\right)}}{{\left(N\right)}^{\left(2i+1\right)}} \end{array}$$

Because *λ* is far less than 1, to guarantee minimum coning error, the low power item of *λ* in Equation (4) should be zero. It is equivalent to make previous *N*+*P*−1 items of the right of Equation (4) to be zero. Letting matrix *F_N_*_+_*_P_*_−1_ represents the previous *N*+*P*−1 item of *F*, we obtain:

(5) $${\text{F}}_{N+P-1}\cdot {\left[\begin{array}{ccccccc} K_{1-P} & K_{2-P} & \cdots & K_{0} & K_{1} & \cdots & K_{N-1} \end{array}\right]}^{\text{T}}={\left[\begin{array}{ccccc} 1 & 1 & 1 & \cdots & 1 \end{array}\right]}^{\text{T}}$$

When *N* and *P* are given, the undetermined coefficient *K⃗* is expressed as:

(6) $${\left[\begin{array}{ccccccc} K_{1-P} & K_{2-P} & \cdots & K_{0} & K_{1} & \cdots & K_{N-1} \end{array}\right]}^{\text{T}}={\text{F}}_{N+P-1}^{-1}\cdot {\left[\begin{array}{ccccc} 1 & 1 & 1 & \cdots & 1 \end{array}\right]}^{\text{T}}$$

Although the simulation results in [22,23] show that two-sample algorithm with *P* = 2 has the highest compensation accuracy, while three-sample algorithm with *P* = 3 has the highest compensation accuracy, experiments suggest that the simulation computation and real test results don't completely correspond to each other because of some small quantity being neglected in the formula derivation [24], especially the effect of the digital filter on the signal of RLGs. Therefore, the selection of compensation parameter should be determined according to practical conditions. Wobbling tests demonstrate that *P* should not be too large, otherwise, overcompensation will possibly occur and this will bring about new errors. In this paper, we set *N* = 2 and *P* = 1. According to Equation (6), we get *K*_1_ = 11/15 and *K*_0_ = 1/30, then the coning errors compensation formula is derived:

(7) $$\Delta \vec{\hat{\Phi }}=\Delta {\vec{\theta }}_{1}+\Delta {\vec{\theta }}_{2}+\frac{11}{15}\Delta {\vec{\theta }}_{1}\times \Delta {\vec{\theta }}_{2}+\frac{1}{30}\Delta {\vec{\theta }}_{1}\times \Delta {\vec{\theta }}_{0}$$

**Figure 1.**
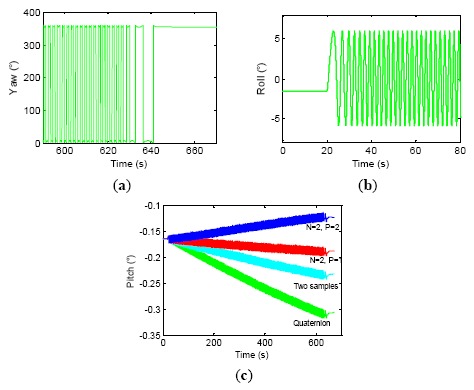
Compensation results of different coning correction algorithm. (**a**) Yaw, (**b**) Roll, (**c**) Pitch.

A two-axis wobbling test was applied to one set of LINS on a wobbler with amplitude 6° and frequency 1 Hz for 10 min. Four kinds of attitude updating algorithms were employed to perform an offline navigation computation, which were respectively quaternion, optimal two-sample, two-sample with one previous sample (*N* = 2, *P* = 1) and two-sample with two previous samples (*N* = 2, *P* = 2). The test results are shown in Figure 1 and Table 1. Figure 1a shows the last 80 s yaw angle curve, Figure 1b shows the first 80 s roll angle curve, and Figure 1c is the pitch curve of the whole experiment process. From the experiment results, we can see that the coning errors emerge on the pitch axis of LINS when two-axis wobbling movement is imposed on the yaw axis and the roll axis simultaneously. A constant angular rate appears on the pitch axis due to the coning errors, as the result, the pitch error drifts linearly. This shows that different attitude updating algorithms have different abilities to reject coning errors in Figure 1, and the traditional quaternion induces the biggest drift error than other algorithms. Table 1 suggests that the four algorithms adopted in the paper generate pitch drift errors of 0.1381°, 0.0646°, 0.0276° and −0.0625°, respectively and the two-sample algorithm with one previous sample (*N* = 2, *P* = 1) is the most precise algorithm in the paper, while the two-sample algorithm with two previous samples (*N* = 2, *P* = 2) produces overcompensation, and it introduces new errors.

According to the experimental results, the two-sample algorithm with one previous sample (*N* = 2, *P* = 1) is employed in this paper. Despite the fact the coning errors are decreased much more by the optimizing compensation algorithm, what we study in the paper is a high precision dynamic attitude system, therefore, the other factors inducing dynamic errors should be reduced as little as possible.

### 2.2. Coning Error Rejection by Enhancing Sampling Frequency

To further reduce coning errors, one of the effective ways is to enhance the attitude updating frequency if the hardware configuration of the navigational computer is fast enough to accommodate this. Besides navigation calculations, the computer of the LINS employed in the experiments is also designed to perform many extra missions in one attitude update interval, such as communication with other devices, inertial sensor temperature measurement and LINS state checking. After neglecting some unnecessary missions, the attitude updating frequency of LINS is increased from 200 Hz to 2000 Hz (but 200 Hz is selected in Section 2.1).

**Figure 2.**
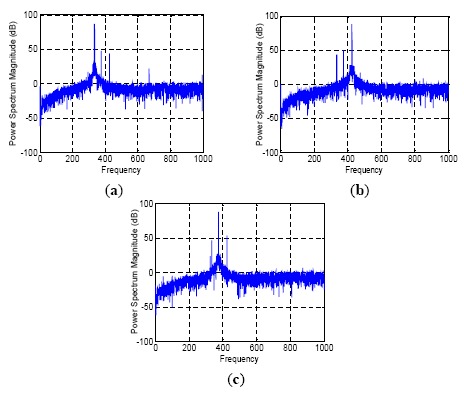
Frequency spectrum of RLGs in LINS. (**a**) Gx, (**b**) Gy, (**c**) Gz.

Subsequently, another problem occurs. Due to the interplay among dither frequencies of the three mechanically dithered RLGs in LINS, there are three dither frequency signals in each gyro output signal. The power spectrum analyses are shown in Figure 2. Three peaks can be obviously found in every figure. This suggests that the RLGs interfere each other intensively when the LINS is working, and the power of the three dither frequencies is all very strong in each gyro signal. Enhancing attitude updating frequency means that the data smoothing time of RLG will be reduced and the capability of rejecting disturbance of dither disturbance will be declined. As the result, the pseudo coning error might form faulty compensation [25] and the oscillation amplitude of attitude algorithm results will be increased. Therefore, if attitude updating frequency is enhanced, the digital filter should be optimized to filter out more dither frequency signals.

In this paper, a special FIR filter with three notch points is configured to aim at the dither frequency points of three RLGs in the LINS. Three dither frequencies are trapped simultaneously when the signals of RLG pass through the filter. The data acquisition frequency of LINS is set at 2000 Hz, and the dither frequencies of three RLGs are 335.4 Hz, 375.5 Hz, and 425.7 Hz, respectively. The FIR filter coefficients are designed as [−3.6162e−004 −5.5582e−005 −4.6181e−004 1.3181e−003 4.3263e−003 1.1456e−002 2.3008e−002 3.9629e−002 6.0210e−002 8.2250e−002 1.0213e−001 1.1603e−001 1.2103e−001 1.1603e−001 1.0213e−001 8.2250e−002 6.0210e−002 3.9629e−002 2.3008e−002 1.1456e−002 4.3263e−003 1.3181e−003 −4.6181e−004 −5.5582e−005 −3.6162e−004], and the bandwidth is kept at 100 Hz.

**Figure 3.**
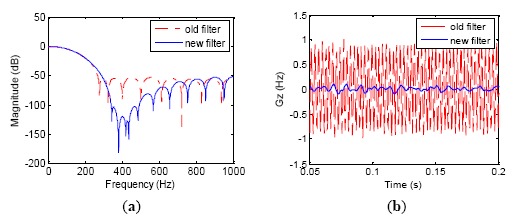
New FIR filter with three notches. (**a**) Amplitude frequency characteristics; (**b**) Effect comparison between new and old filter.

Figure 3a shows the amplitude-frequency characteristic of the filter. The amplitude-frequency curves of the old and new filter coincide with each other under 150 Hz, meaning that the bandwidth of the two FIR filters is same. Besides three notch points to trap the dither frequencies, the new filter provides extra attenuation at least 50 dB in the frequency band from 320 Hz to 420 Hz to further decay the dither signals. Consequently, the performance of the new filter is always efficient although the dither frequencies might slightly change after the LINS works for a long time. Figure 3b shows the filtering effect comparison between old and new filters. Because three dither frequencies are notching filtered by the new filter, the gyro output signal fluctuation is decreased five times compared to the previous results.

Another reason for selecting a FIR filter in the paper is that the FIR filter has a linear phase, which will guarantee all frequency signals have the same time delay after passing the filter. This ensures that the phase compensation mechanism will be effective for all frequencies.

### 2.3. Experimental Verification of Coning Errors Compensation Effect

After above improvements, three-axis wobbling test was applied to verify whether the accuracy of the LINS dynamic attitude was enhanced. The experiments used an old-fashioned wobbler, which was unable to output three gimbals angles and could not return to initial position after completion of wobbling tests. Therefore, the wobbler could not provide any message to evaluate the accuracy of the LINS. In fact, LINS generally has higher horizontal initial alignment precision and lower azimuth alignment precision. If the horizontal attitudes accuracy is high enough, the pitch and the roll angle can be uesed as the reference. According to the theoretic limits accuracy calculation formula [11]:

$$\psi =\frac{{ɛ}_{e}}{{\text{ω}}_{ie}\cos L},\gamma =\frac{\nabla_{e}}{g},\theta =\frac{\nabla_{n}}{g}$$

where *Ψ* is azimuth alignment error, *ε_e_* is east gyro biasstability, *ω_ie_* is rotate rate of the earth, *L* is latitude, *γ* and *θ* are roll and pitch alignment error respectively, ∇*_e_* and ∇*_n_* are east and north accelerometer bias stability, *g* is acceleration of gravity.

The high precision LINS used in the experiments was composed of accelerometers and RLGs with bias stability of 2 × 10^−5^ *g* and 0.005°/*h*, respectively Theoretically, its horizontal alignment accuracy was 4″, but its azimuth alignment accuracy was about 2′ in Xi'an, China. Thus, the paper employed the difference between previous navigation results and next alignment results of two horizontal attitudes as the error criterion.

Three groups of wobbling tests were performed, where the experimental conditions were set at: 10 min in testing time, 7° in amplitude, 0.5 Hz in frequency. The attitude updating frequency was set at 2000 Hz. The wobbler remained static for 300 s∼400 s at the beginning and at the end of the test process, and both the static data were used for initial alignment. The first alignment provided the initial attitudes for the first navigation, and the second alignment made use of the comparison with the results of the first navigation, and it can also provide initial attitudes for the second navigation. The detailed process was as follows: after initial alignment, the wobbler performed three-axis wobbling movement for about 10 min. The position was kept unchanged after the wobbler was stopped, 5 min data of LINS collection continued to be executed and these data were used to carry out the next alignment. Ideally, the navigational results of pitch and roll angles should be equal to those obtained in the second initial alignment, otherwise errors would be induced.

**Figure 4.**
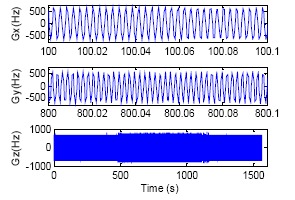
Gyro raw data during three-axis wobbling.

**Figure 5.**
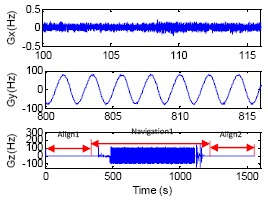
Processing by new filter.

**Figure 6.**
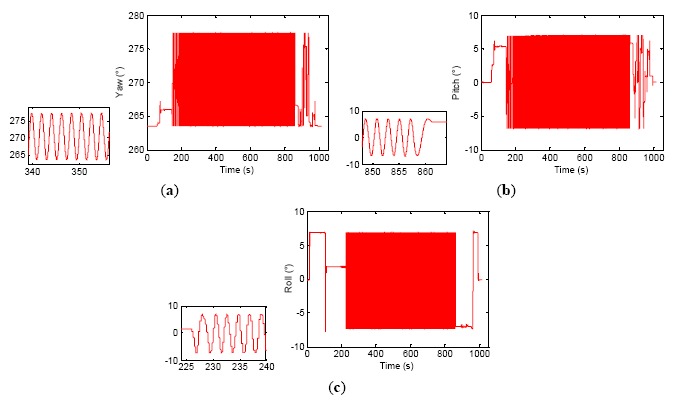
LINS attitudes output during wobbling test (2000 Hz attitude update rate). (**a**) Yaw, (**b**) Pitch, (**c**) Roll.

The experimental results are shown in Figures 4, 5 and 6 and Table 2. In Figure 4, three gyro raw pulse data curves for different test time segments are shown, respectively. The Gx curve shows the original gyro X output when the wobbler remains static, the Gy curve is the gyro Y output when the wobbler is in movement and Gz presents the whole test process. The magnitude of RLG raw data reaches as high as ±600 Hz/0.5 ms in Figure 4 due to the dither interference, even if under static circumstances.

After the LINS is excited by 7° and 0.5 Hz wobbling movement, the fluctuation range of the gyros data is up to ±750 Hz/0.5 ms; this point can be seen more clearly from the Gz curve in Figure 4. The interference of dither frequencies is mostly eliminated after the three sets of gyro raw data are processed by the digital filter proposed by this paper, and the results are shown in Figure 5. It is suggested by the Gx curve that the gyro output is within ±0.2 Hz/0.5 ms when the wobbler remains static. A clear sinusoidal movement trail can be found from the Gy curve during the wobbling test. The two alignment and navigation processes are shown in the Gz curve in Figure 5. The alignment and navigational calculation are executed by using the data output from the digital filter, and the result is shown in Figure 6. In order to be clear, both the whole process and the partial detail attitude curves of the LINS during the wobbling test are shown in Figure 6. As mentioned before, the difference of level attitudes between the first navigation and second alignment is adopted as the accuracy criterion. The second alignment result should have the same attitude as the first navigation because the wobbler is kept static. The data provided in Table 2 are the first navigation results minus the second alignment results. Clearly, the attitude accuracy of LINS is further enhanced after the sampling frequency is increased, and the maximum error is better than 0.02°.

## 3. Time Synchronization

### 3.1. Analysis of Phase Lag of LINS

According to automation and signal processing theory, if a signal passes through a system, the system will introduce an extra phase shift in the output signal. For example, after an analog signal passes a RC filter, the filter will not only decrease the high frequency noise but also produce a phase lag compared with the original signal. Similarly, for digital systems such as digital controllers, digital filters and digital signal processing algorithms will also cause an extra phase delay. There are data acquisition, digital filters and other elements in the LINS, therefore the LINS navigation output results are bound to lag behind the input stimulation signals. In a dynamic attitude test, the phase lag would result in measurement errors whose expression is given by the following theorems:

*Theorem 1*: When an INS is excited by a low-frequency sinusoidal simple harmonic oscillation, after navigational calculation, the maximum attitude measurement error induced by phase lag is expressed as:

(8) $$\mathrm{Max}(\text{erro}r_{\text{att}}(\omega ))=A\Delta \eta (\omega )$$

where, Δ*η*(*ω*) represents phase-frequency characteristic of transfer function of INS sensing axis. The amplitude and frequency are represented by *A* and *ω* respectively.

*Proof*: Assume the attitude excitation on the INS is as follows:

(9) $$y_{i}(t)=A_{i}\sin(\omega_{i}t+\eta_{0i})i=\psi ,\theta ,\gamma$$

where *ψ, θ* and *γ* represent the heading, pitch and roll angles, respectively, *ω_i_* is the attitude angular rate, *A_i_* is the attitude angle amplitude, *η*_0_*_i_* is the initial phase and *t* is time. After attitude calculation by the INS, the following attitude angles are obtained:

(10) $${y^{\prime }}_{i}(t)={A^{\prime }}_{i}(\omega_{i})\sin(\omega_{i}t+\eta_{0i}+\Delta \eta (\omega_{i}))i=\psi ,\theta ,\gamma$$

where, *A*′*_i_*(*ω_i_*)=|*G_i_*(*jω_i_*)|*A_i_* is the INS attitude angle amplitude output, Δ*η*(*ω_i_*) = ∠*G_i_*(*jω_i_*) is the attitude angle phase lag induced by the INS, and *G_i_*(*s*) is the transfer function of each attitude channel of the INS. Therefore, dynamic attitude measurement errors are defined as:

(11) $$\text{erro}r_{i}=y_{i}(t)-{y^{\prime }}_{i}(t)=A_{i}\sin(\omega_{i}t+\eta_{0i})-{A^{\prime }}_{i}(\omega_{i})\sin(\omega_{i}t+\eta_{0i}+\Delta \eta (\omega_{i}))$$

At the low frequency band, |*G_i_*(*jω_i_*)| ≈ 1, *A*′*_i_*(*ω_i_*) ≈ *A_i_* and Δ*η*(*ω_i_*) is a small angle, then:

(12) $$\text{erro}r_{i}=-A_{i}\Delta \eta (\omega_{i})\cos(\omega_{i}t+\eta_{0i}+\Delta \eta (\omega_{i})/2)$$

therefore:

(13) $$\mathrm{Max}(\text{erro}r_{\text{att}}(\omega ))=A_{i}\Delta \eta (\omega_{i})$$

Because Δ*η*(*ω_i_*) is very small, the effect of Δ*η*(*ω_i_*) can be neglected, and the dynamic attitude measurement errors then become:

(14) $$\text{erro}r_{i}\approx -A_{i}\Delta \eta (\omega_{i})\cos(\omega_{i}t+\eta_{0i})$$

From Equations (12)–(14), we can arrive at the following conclusions:

(a) If INS is an input sinusoidal angle rate exciting signal, the dynamic attitude errors of the INS output should a cosine signal with same frequency, and its amplitude is *A_i_*Δ*η*(*ω_i_*).
(b) According to Equation (14), the dynamic attitude measurement error lags the input exciting signal by π/2 in the low frequency band.
(c) The error of dynamic attitude measurement amplitude is both proportional to the phase lag of INS and the amplitude of the exciting signal.

Because INS works in a real time model, it is more convenient to compensate the INS lag in the time domain. Δ*η*(*ω_i_*) can be represented as in the time domain:

(15) $$\Delta \eta (\omega_{i})=\omega_{i}t_{d}$$

where, *t_d_* is the INS lag time. Substituting Equation (15) into Equation (13), we can obtain another expression of the maximum attitude measurement error:

(16) $$\mathrm{Max}(\text{erro}r_{\text{att}}(\omega_{i}))=A_{i}\omega_{i}t_{d}$$

It further suggests that the maximum INS attitude measurement error is proportional to *A_i_, ω_i_* and *t_d_*. The amplitude *A_i_* and frequency *ω_i_* cannot be changed because the input signal is stochastic and it comes from dynamic maneuver environments. Thereby, diminishing or eliminating the time lag of LINS *t_d_* is another key point to decrease LINS dynamic measurement errors. Furthermore, if *t_d_* can be estimated and compensated completely (*i.e., t_d_* = 0), we can obtain *Max*(*error_att_*(*ω_i_*)) = 0, which means that INS can be used to measure the dynamic attitude without errors introduced by time delay.

However, the phase Δ*η*(*ω_i_*) or the time lag of an INS are inherent to its mechanical structure, the characteristics of the gyro, and the methods of data acquisition and processing. A RLG has a wide dynamic measuring range compared with traditional gyros because the RLG has no mechanical rotor. Consequently, LINS is suitable for dynamic maneuvering tests. In order to reduce the phase lag of LINS attitude, the factors producing the lag are analyzed as follows:

(a) *The RLG phase lag*. It is pointed out in [26] that the RLG bandwidth itself exceeds 30 kHz. Also in [27], it is believed that the RLG bandwidth is mainly restricted by the readout circuits, and that the real bandwidth of the RLG will be above 1000 Hz in practical applications, even with mechanical configuration restrictions on it, and in [27] it is further indicated that the signal delay due to RLG is only 5 μs in aircraft applications. Generally, the INS bandwidth used in the airplane is less than 100 Hz, which corresponds to a useful signal sampling period of 10 ms. In view of this, the time lag caused by the RLG itself is considered negligible in this paper.
(b) *The speed of readout circuits*. Presently, a high-speed CPLD is commonly adopted in readout circuits. Its set-up time is measured in picoseconds [28], and its readout time is several microseconds, which can also be negligible.
(c) *The digital signal processing*. According to what has been mentioned above, the 24 order FIR filter with three notch points is adopted by the paper. Its group delay is 12. The time delay is 6 ms if the sampling frequency is 2 kHz. The value of the time delay is a constant because the FIR filter has a linear phase for all frequencies. This is of great importance for phase compensation.
(d) *Data acquisition and updating will also induce delays*. After the navigation computer gathers the frequency signal from RLGs, it will execute navigation calculations; the procedure is equivalent to a zero-order holder. Its transfer function is as follows:
  (17) $$G(j\omega )=\frac{T\sin(\omega T/2)}{\left(\omega T/2\right)}e^{-j\omega T/2}$$
  where, *T* is sampling time, and *T* = 0.5 ms. The time delay is *T*/2 when angular frequency *ω* is equal to sampling frequency *ω_s_*. Therefore the time delay due to data acquisition and navigation calculation is 0.25 ms.

According to the above analysis, the time delay of the LINS is an approximately fixed value. Theoretically, the attitude angle output from the LINS lags the real attitude input by 6.25 ms. The key idea in the paper is to transform a variable of phase lag in the frequency domain into a fixed time delay in the time domain.

But how to prove the ideas proposed by the paper are correct? How to design a synchronization clock to compensate the time delay of LINS? What kind of equipment can be used as a reference to judge the LINS dynamic attitude measurement accuracy? All these questions are new problems because documentation about the case can seldom be found.

In this paper, we want to develop a kind of synchronization mechanism which can make two or more devices have rigorous correspondence at every test time point. Furthermore, the phase relationship of those devices can be compensated by the synchronization mechanism.

### 3.2. Design and Experimental Verification of Synchronization Mechanism

**Figure 7.**
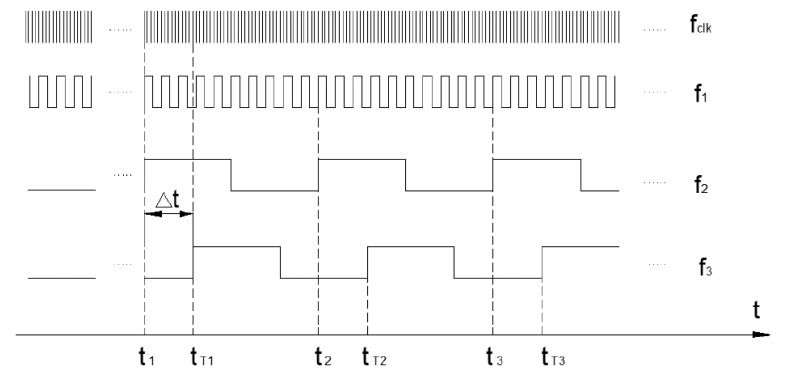
Time sequence of synchronization mechanism.

In order to reduce the dynamic errors due to phase lag of the LINS, a novel synchronization mechanism is devised to change the phase relationship between the input exciting signal of the turntable and the attitude output of the LINS based on the state machine in a CPLD. The time sequence is illustrated in Figure 7.

Assuming that clock frequency of the navigation computer interrupt is *f_clk_*, through frequency division of *f_clk_*, the initial signal acquisition frequency of the gyro and the operating frequency of the FIR filter is 2 kHz, the attitude updating calculation frequency is 2 kHz too. The original attitude output frequency is *f*_1_ = 1 kHz because the coning compensating algorithm employs two-sample with one previous sample algorithm (*N* = 2, *P* = 1). Usually, 1 kHz attitude output frequency is too high for users to record and analyze data, so the real attitude output frequency of LINS is down-sampled to *f*_2_ = 100 Hz. The attitude data of LINS will be latched by the rising edge of *f*_2_ on the moment of *t*_1_, *t*_2_, *t*_3_,…, and the data are regarded as the LINS attitude measuring results. The latch signal *f*_3_ does not need to be sent to other devices requiring synchronization (for example, a wobbler or a turntable) until the signal is delayed by a time Δ*t*. The attitude angle (or the gimbal angle of turntable) will be latched by the rising edge of the synchronization signal on the moment *t_T_*_1_, *t_T_*_2_, *t_T_*_3_,… By means of asynchronous latching of two or more different devices according to the synchronization signal of LINS, the rigorous time correspondence between two or more groups of data will be assured. According to Section 3.1, the theoretic value of Δ*t* is 6.25 ms, but to make up for the time delay in the data latch of the wobbler and some unconsidered facts, the value of Δ*t* is designed as a variable which can be fine-adjusted.

**Figure 8.**
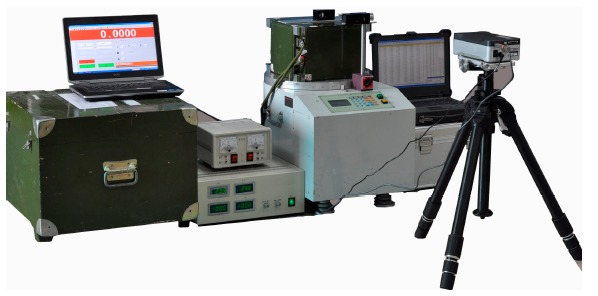
Synchronization mechanism verification equipment.

**Figure 9.**
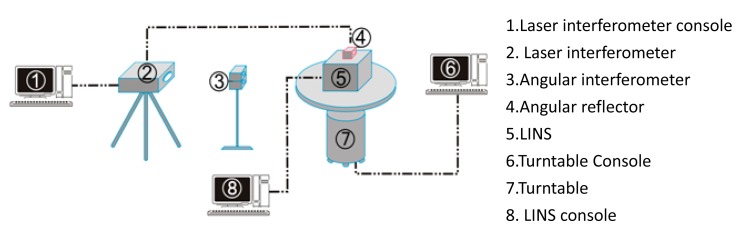
Connection of synchronization mechanism verification equipments.

The aforementioned synchronization mechanism was put forward for the first time by the authors. Some methods should be found to verify its effectiveness. A laser interferometer (XL-80, RENISHAW Company, Gloucestershire, UK) was used as a reference device, and a single-axis turntable was employed to provide an azimuth sinusoidal exciting signal. The test setup was configured as seen in Figure 8 and the connection diagram is shown in Figure 9. According to the user's manual of the laser interferometer [29], the angle measurement accuracy of the RENISHAW XL-80 interferometer was 0.5″ by using angular measurement optics, or high enough to be a reference device for the LINS in this paper.

The turntable was leveled before the examination. The LINS was put on the turntable and the angular reflector was fixed on the LINS in the tests. After initial alignment, the single-axis turntable was controlled to perform wobbling movement. The LINS made real-time measurements of the angle output from the turntable, and sent a synchronous pulse *f*_3_ to the laser interferometer which latched and recorded the angle at the moment of the rising edge of *f*_3_. The output frequency of both devices is 100 Hz, so the same two groups of data will be obtained during the test.

It should be emphasized that the output angle meanings of the two devices were different. What the laser interferometer recorded was the relative angle rotated by the turntable, while the azimuth angle output from the LINS was associated with the geographic coordinate system, and only when the data of the two groups were associated with the same coordinate frame, would there be a contrast between them. The pretreatment method in the paper was all the data of LINS azimuth angle were subtracted from its initial alignment results, and the relative angular displacement based on the initial alignment angle could be obtained. After the preprocessing, the two groups of the data had same frame definition and a comparison could be made. The test time was about 50 s, and 5000 data points could be obtained from these two devices, respectively. Making a one-to-one subtraction to these two groups of data, and we would get the errors, which were defined in Equation (11).

In terms of the above method, the dynamic measurement errors of the general LINS were tested first. In this case, the time delay compensation parameter Δ*t* was set to zero. Selecting the condition of wobbling movement at 7° in amplitude and 0.5 Hz in frequency, according to above, the LINS had a time lag *t_d_* = 6.25 ms, and it would produce a theoretical phase lag given by:

(18) $$\Delta \eta (\omega_{i})=\omega_{i}t_{d}=2\times \pi \times 0.5\times 0.00625=1.9635\times 10^{-2}(\text{rad})$$

From Theorem 1, the theoretical amplitude of dynamic measurement error induced by the LINS phase lag was:

(19) $$\Delta \eta (\omega_{i})\times 7{}^{\circ}=0.1374{}^{\circ}$$

**Figure 10.**
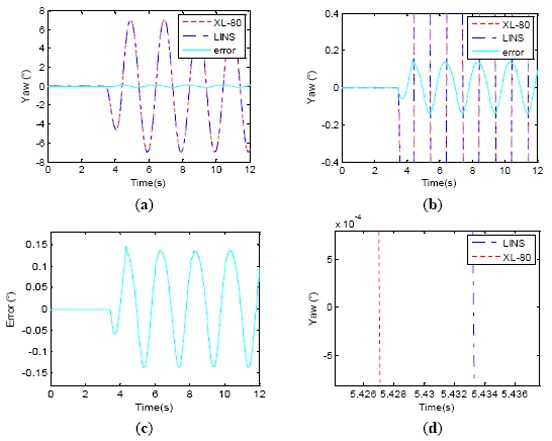
Wobbling test results without time delay synchronization. (**a**) First 12 s curve of wobbling test; (**b**) Phase of error lag turntable *π*/2; (**c**) Error curve; (**d**) Phase relationship between table and LINS.

The verification test results with Δ*t* = 0 are shown in Figure 10. In Figure 10a, the data curves of the LINS and laser interferometer in the first 12 s of the wobbling test are shown. The error curve of the differences between corresponding points of the LINS and laser interferometer are also shown in Figure 10a. Figure 10b is the partially magnified error curve of Figure 10a; it shows that the error curve lags the single-axis turntable wobbling signal by π/2, which indicates that if the LINS input is a sinusoidal exciting signal, the LINS dynamic error is a negative cosine signal, just as expressed by Equation (14). The dynamic errors are shown in Figure 10c, which suggests that the amplitude of the error curve is 0.1368° while its theoretical value is 0.1374°, and their difference is only 6 × 10^−4^°. From Figure 10d, we could estimate that the LINS output curve is delayed 6.22 ms compared with that of laser interferometer, while its theoretical value is 6.25 ms. The test results demonstrate that the Theorem 1 and Equations (8)–(14) derived in this paper are correct. The standard deviation of the LINS dynamic attitude measurement error is 0.097°. It is just a usual LINS accuracy. The experimental results suggest that the phase lag of LINS indeed brings about greater error for dynamic attitude measurement. Consequently, the general LINS without phase compensation is not suitable for high precision dynamic attitude measurements.

**Figure 11.**
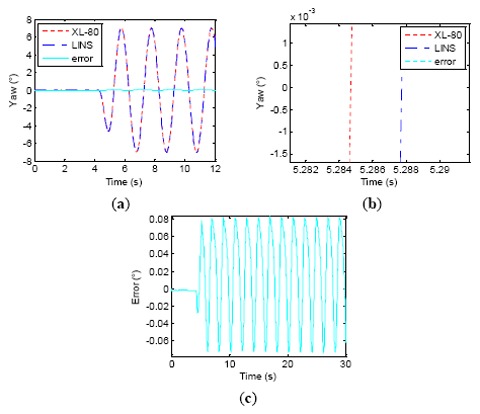
Δ*t* = 3 *ms* wobbling test results. (**a**) First 12 s wobbling test; (**b**) Phase relationship between table and LINS; (**c**) Error curve.

**Figure 12.**
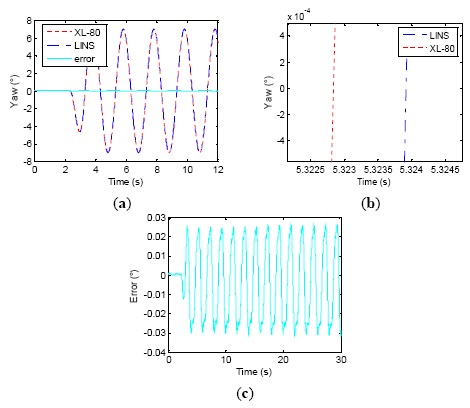
Δ*t* = 5 *ms* wobbling test results. (**a**) First 12 s wobbling test; (**b**) Phase relationship between table and LINS; (**c**) Error curve.

**Figure 13.**
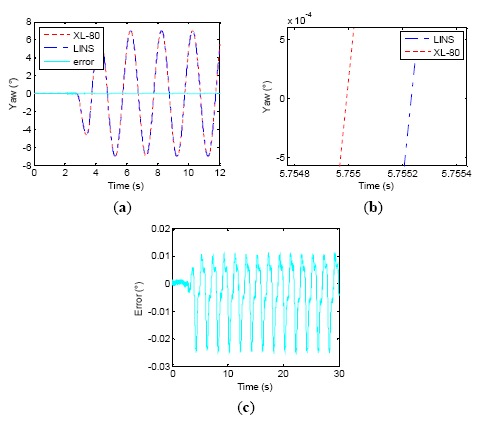
Δ*t* = 6.1*ms* wobbling test results. (**a**) First 12 s wobbling test; (**b**) Phase relationship between turntable and LINS; (**c**) Error curve.

In the subsequent tests, we selected different Δ*t* values, which were 3, 5 and 6.1 ms, shown separately in Figures 11, 12 and 13, to verify whether or not the phase relations between the attitude curves from turntable and LINS could be changed.

**Figure 14.**
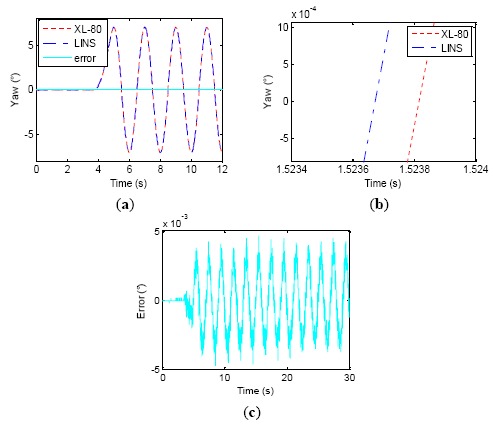
Δ*t* = 6.25*ms* wobbling test results. (**a**) First 12s wobbling test; (**b**) Phase relationship between turntable and LINS; (**c**) Error curve.

We can see that as Δ*t* grows, the phase difference between attitude curves of the laser interferometer and LINS gradually decrease. Therefore, the amplitude of attitude angle errors is decreased, and the attitude measurement accuracy is improved little by little. When Δ*t* = 6.1 ms, the LINS output stably delays the laser interferometer result by about 0.2 ms. However, as Δ*t* progressively approximates its theoretical value of 6.25 ms, the phase relationship between LINS and laser interferometer becomes stochastic, and the phase of the LINS yaw sometimes exceed that of the laser interferometer. Figure 14 shows one test result for this kind of case, where the phase of the LINS yaw leads that of the laser interferometer by 0.13 ms. The reasons behind this phenomenon are some subsidiary facts that are neglected when analyzing the LINS phase lag, such as the stochastic error of RLG, the characteristics of the rubber damper and the latching delay of the laser interferometer. All of them can also induce a certain phase lag. Table 3 shows the change process of the dynamic attitude measurement errors of the LINS and the remainder delay time. When Δ*t* is increased from 0 to 6.1 ms, the lag time of the LINS is reduced from 6.22 ms to 0.27 ms, and the accuracy is improved from 0.097° (1σ) to 0.011° (1σ). When Δ*t* = 6.25 ms, the accuracy of LINS is enhanced to 0.003° (1σ). The experiments demonstrate that the synchronization method proposed by the paper is effective, and the phase relationship between two types of equipment can be adjusted by changing the value of Δ*t*, which has a noticeable effect in enhancing the dynamic attitude measurement accuracy of LINS, and the time synchronization accuracy is about 0.2 ms by using the described experimental equipment.

## 4. Dynamic Attitude Measurement Accuracy Tests of DAMS

### 4.1. DAMS Hardware Configuration

**Figure 15.**
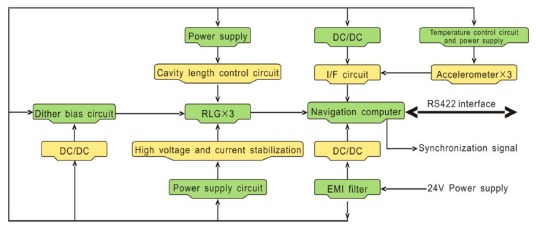
DAMS composition.

**Figure 16.**
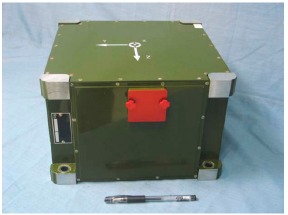
DAMS photo.

With the adoption of improved methods put forward in this paper, a high precision LINS dedicated to dynamic attitude measurement is developed, called dynamic attitude measurement reference system, abbreviated as DAMS. In the system, the angular rate measurement channels are constituted by three RLGs, frequency stabilization control circuit, dither control circuit and high voltage stabilization current circuit, and the acceleration measurement channels are constituted by three quartz accelerometers, I/F conversion circuit and accelerometer temperature control circuit. A TMS320C6713 is selected as the solution platform of the navigation computer. Both inner sampling frequency and attitude updating frequency are set at 2000 Hz, but the data output frequency is adjusted down to 100 Hz because the data are too much for the computer to receive and record as mentioned before. The communication interface is a RS422 serial port, whose baud rate is 921.6 kb/s. Another special interface is the synchronization signal, which is used to compensate the time delay of the DAMS. A diagram of the DAMS is shown in Figure 15. The performance parameters of the RLGs and accelerometers employed are listed in Table 4, and a real photo is shown in Figure 16.

### 4.2. Dynamic Attitude Accuracy Measurement Test

**Figure 17.**
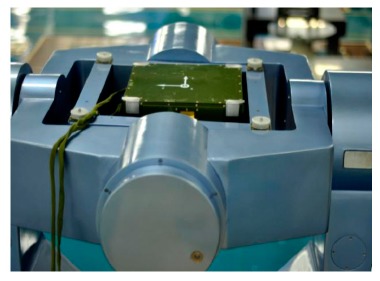
Picture of DAMS mounted on the three-axis turntable.

**Figure 18.**
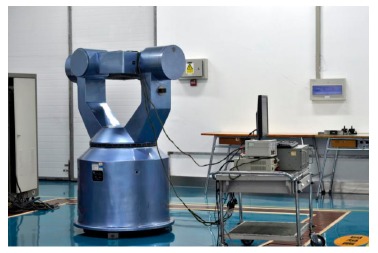
Picture of the whole experimental layout.

The experiments employed a three-axis turntable with synchronization function, which could latch three gimbal angles after receiving the synchronization signal. The DAMS mounted on the inner gimbal of the three-axis turntable is shown in Figure 17. Figure 18 presents the whole experiment layout. After initial alignment, the DAMS executed the attitude updating algorithm and started to output pitch, roll, azimuth angle and simultaneously sent the synchronization frequency signal to the console of the turntable at 100 Hz. The turntable performed three-axis wobbling excitation of the DAMS. After receiving the synchronization signal sent by the DAMS, the console of the turntable latched the three gimbal angles. Two experimental conditions were selected according to the different amplitude and frequency (see Table 5). The experiments were carried out three times under every condition, and in all the experiments the test time was 10 min. Because of the motor power restriction, the amplitude and the frequency of the external gimbal were lower than that of the middle and the inner gimbal. In the experiments, the wobbling amplitude had to decline as the wobbling frequency increased because of the limited motor power and dynamic characteristics, and the highest frequency could just reach 2 Hz.

The gimbal angles of the three-axis turntable were regarded as the reference of the DAMS when the data were processed, but the gimbal angles and DAMS output attitude angles had different zero base definitions. As mentioned above, it was necessary to modify them to the same coordinate system to evaluate the DAMS errors. By subtracting the initial alignment results from the DAMS output attitude angle, we obtained the relative pitch, roll and azimuth angle. Likewise, by subtracting the initial gimbal angles from the three-axis turntable outputs, we got the relative gimbal angles. Through such data pre-processing and one-to-one subtraction of these two groups of data, we could obtain the dynamic errors of the DAMS, and the standard deviation of the errors was considered as the final accuracy criterion. The test results are listed in Table 6.

**Figure 19.**
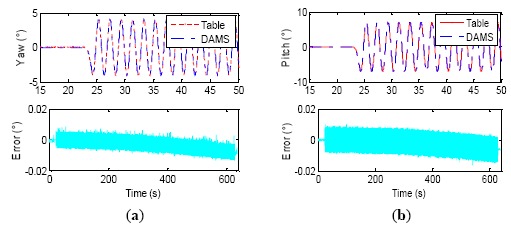

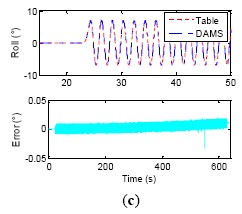
Three axes wobbling test process and error curves (0.5 Hz). (**a**) Yaw, (**b**) Pitch, (**c**) Roll.

**Figure 20.**
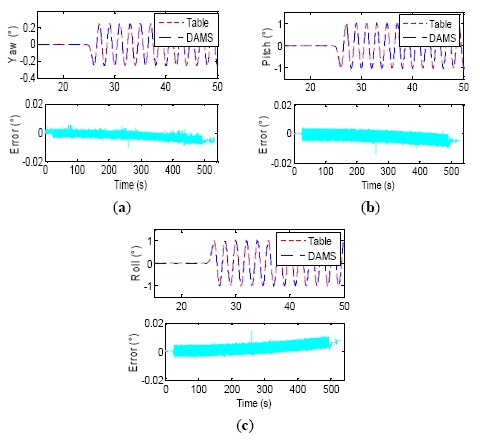
Three axes wobbling test process and error curve (2 Hz).

It can be seen from Table 6 that a small residual phase lag still exists in the DAMS output although it has been compensated and this residual time lag is about 0.2 ms (except for No. 2, where it is up to about 0.35 ms). The phase relationship between two devices is stochastic too, and the attitude angles of DAMS possibly lead the gimbal angles of the turntable. The dynamic attitude measurement accuracy of the DAMS is about 20″ (1σ) after the synchronization compensation proposed by the paper. Figure 19 illustrates the experiment process of the second group data, with the maximum synchronization error in Table 6. The test curves of yaw, roll and pitch within 15–50 s are shown in Figure 19, and the error curves are for the whole experimental process. The three attitude errors drift approximately linearly, but their drift rate has already become very slow, about 1 × 10^−5^ °/s, which demonstrates that coning error algorithm has a better compensation effect. One test result of the second wobbling condition (the 4th group of data in Table 6) is illustrated in Figure 20, where the wobbling frequency is improved to 2 Hz, and the DAMS could measure and trace the signal of the turntable too. The errors of dynamic attitude measurement are less than 0.01°, suggesting that the dynamic wobbling errors at 2 Hz have equivalent magnitude and drift speed as those at 0.5 Hz.

In the end, it should be pointed out that the gimbal angles of the three-axis turntable are selected as the reference in order to evaluate the dynamic attitude measurement accuracy of the DAMS. Because of the different coordinate systems, it is necessary to convert the geographical coordinate system of the attitude angle output from the DAMS into a kind of relative coordinate system for comparison purposes, which means the test results in this paper are a relative accuracy, not the true attitude accuracy, especially for the azimuth. Essentially, the DAMS is still a set of high precision LINS, therefore, it can output attitude angles defined in the navigation coordinate system, and its real attitude accuracy is restricted by the accuracy of the RLGs and accelerometers. Compared with a conventional high precision LINS, the DAMS produces less additional dynamic attitude errors under dynamic conditions, and thus can be used in any field which needs dynamic, real time, high precision attitude measurements.

## 5. Conclusions

This paper utilizes a high precision LINS to develop a system that could measure the three attitude angles of a moving base with high precision, that is, DAMS. Compared with conventional LINS, DAMS has many optimizations such as a high precision attitude updating algorithm, improved digital filter for RLG dithering frequencies, high frequency of attitude updating, special synchronization and phase compensation functions. Because of these improvements, DAMS has the ability to measure the real-time dynamic attitude with high precision. For the first time, this paper derives the theoretical formula of attitude measurement error induced by the INS phase lag, and proposes a new verification method which adopts a single-axis turntable and laser interferometer to confirm the correctness of this derivation. The experiments of the three-axis wobbling tests demonstrate that the dynamic attitude measurement accuracy of DAMS can reach up to about 20″, and the time synchronization accuracy can reach up to about 2 × 10^−4^ s. As a higher precision attitude reference device, DAMS could be applied in any sensor fusion field requiring rigorous space attitudes and time synchronization, and also in calibrating dynamic errors of other attitude measurement systems.

## Figures and Tables

**Table 1.** Comparison of the accuracy of the four attitude updating algorithms.

**Algorithm**	**Attitude Updating Formula**	**Coning Error (°)**
Quaternion	$\Delta \vec{\hat{\Phi }}=\Delta {\vec{\theta }}_{1}$	0.1381
Two samples	$\Delta \vec{\hat{\Phi }}=\Delta {\vec{\theta }}_{1}+\Delta {\vec{\theta }}_{2}+\frac{2}{3}\Delta {\vec{\theta }}_{1}\times \Delta {\vec{\theta }}_{2}$	0.0646
*N* = 2, *P* = 1	$\Delta \vec{\hat{\Phi }}=\Delta {\vec{\theta }}_{1}+\Delta {\vec{\theta }}_{2}+\frac{11}{15}\Delta {\vec{\theta }}_{1}\times \Delta {\vec{\theta }}_{2}-\frac{1}{30}\Delta {\vec{\theta }}_{1}\times \Delta {\vec{\theta }}_{0}$	0.0276
*N* = 2, *P* = 2	$\Delta \vec{\hat{\Phi }}=\Delta {\vec{\theta }}_{1}+\Delta {\vec{\theta }}_{2}+\frac{323}{420}\Delta {\vec{\theta }}_{1}\times \Delta {\vec{\theta }}_{2}-\frac{13}{210}\Delta {\vec{\theta }}_{1}\times \Delta {\vec{\theta }}_{0}+\frac{1}{140}\Delta {\vec{\theta }}_{1}\times \Delta {\vec{\theta }}_{-1}$	−0.0625

**Table 2.** Level attitude errors of wobbling test.

**No**	**Error of Roll (°)**	**Error of Pitch (°)**
1	0.0016	−0.0010
2	−0.0095	0.0187
3	0.0056	0.0160

**Table 3.** Relationship between residual lag time and dynamic attitude errors.

**No**	**Δ*_t_* (ms)**	**Lag Time (ms)**	**1σ (°)**
1	0	6.22	0.097
2	3	3.21	0.053
3	5	1.20	0.021
4	6.1	0.27	0.011
5	6.25	×	0.003

**Table 4.** Gyro and accelerometer parameters.

**Accelerometer Errors**	
Bias (micro g)	2.0
Bias stability 1σ (g)	2 × 10^−5^
Scale Factor (ppm)	5.0
Nonorthogonality (arc sec)	1.0

**Gyro Errors**	

Angle Random Walk ( ${}^{\circ}/\sqrt{h}$)	0.0008
Bias (°/*h*)	0.01
Bias stability 1σ(°/*h*)	0.005
Scale Factor (*ppm*)	2.0
Nonorthogonality (arc sec)	1.0

**Table 5.** DAMS three-axis wobbling test conditions.

**NO**	**Inner Gimbal (Roll)**	**Middle Gimbal (Pitch)**	**External Gimbal (Yaw)**
1	7°, 0.5 Hz	7°, 0.5 Hz	4°, 0.5 Hz
2	1°, 2 Hz	1°, 2 Hz	0.25°, 2 Hz

**Table 6.** DAMS three axis wobbling test results (10 min).

**No.**	**Condition**	**Yaw**		**Pitch**		**Roll**	

		**Error**	**Delay Time**	**Error**	**Delay Time**	**Error**	**Delay Time**
		**(″, 1σ)**	**(s)**	**(″, 1σ)**	**(s)**	**(″, 1σ)**	**(s)**
1	1	7.34	−1.44 × 10^−4^	9.94	−1.56 × 10^−4^	−10.34	−1.36 × 10^−4^
2	1	12.74	3.55 × 10^−4^	19.44	3.43 × 10^−4^	20.74	3.63 × 10^−4^
3	1	7.78	−1.44 × 10^−4^	9.91	−1.46 × 10^−4^	10.58	−1.37 × 10^−4^
4	2	14.69	2.61 × 10^−4^	11.35	2.42 × 10^−4^	18.47	2.74 × 10^−4^
5	2	9.66	2.45 × 10^−4^	13.87	2.17 × 10^−4^	15.23	2.35 × 10^−4^
6	2	13.56	−1.56 × 10^−4^	10.43	−1.38 × 10^−4^	12.12	−1.67 × 10^−4^
